# Why Build a Robot With Artificial Consciousness? How to Begin? A Cross-Disciplinary Dialogue on the Design and Implementation of a Synthetic Model of Consciousness

**DOI:** 10.3389/fpsyg.2021.530560

**Published:** 2021-04-21

**Authors:** David Harris Smith, Guido Schillaci

**Affiliations:** ^1^Communication Studies and Media Arts, McMaster University, Hamilton, ON, Canada; ^2^Department of Excellence in Robotics & AI, Scuola Superiore Sant'Anna, Pisa, Italy; ^3^The BioRobotics Institute, Scuola Superiore Sant'Anna, Pisa, Italy

**Keywords:** artificial consciousness, synthetic consciousness, robotics, art, interdisciplinary dialogue, synthetic phenomenology

## Abstract

Creativity is intrinsic to Humanities and STEM disciplines. In the activities of artists and engineers, for example, an attempt is made to bring something new into the world through counterfactual thinking. However, creativity in these disciplines is distinguished by differences in motivations and constraints. For example, engineers typically direct their creativity toward building solutions to practical problems, whereas the outcomes of artistic creativity, which are largely useless to practical purposes, aspire to enrich the world aesthetically and conceptually. In this essay, an artist (DHS) and a roboticist (GS) engage in a cross-disciplinary conceptual analysis of the creative problem of artificial consciousness in a robot, expressing the counterfactual thinking necessitated by the problem, as well as disciplinary differences in motivations, constraints, and applications. We especially deal with the question of why one would build an artificial consciousness and we consider how an illusionist theory of consciousness alters prominent ethical debates on synthetic consciousness. We discuss theories of consciousness and their applicability to synthetic consciousness. We discuss practical approaches to implementing artificial consciousness in a robot and conclude by considering the role of creativity in the project of developing an artificial consciousness.

## 1. Why Build an Artificial Consciousness?

### 1.1. DHS

Human culture owes much to the wish to animate matter, since we are largely constituted in our abilities and status in the world by investing the world with anthropomorphic meaning and agency far into our prehistory (Mithen and Morton, 1996). From stone, bone, and pigments, to writing and print, to sound, image and cinema, to artificial agents, one can trace a progressive anthropomorphic investment in our symbolic technologies, which are now capable of materializing and automating our imaginations, our words, our stories, our storytellers, our conviviality, and our intelligence. It is difficult to imagine that this trajectory will suddenly be arrested. Given the centrality of innovative anthropomorphism to cultural progress, the technical investment of consciousness appears inevitable. But, to what end? What artistic uses can be made of artificial consciousness, especially in the context of robots?

To consider this question, there is an important distinction to be made between the actual realization of sentience in robots and the works, stories, and myths, about sentient robots. There are numerous examples of the latter dating at least from the myth of Talos (700 B.C.) to contemporary films, such as Alex Gardner's Ex-Machina (Gardner, 2014). Wherever, robot artworks have been physically created with phenomenological premises, traits associated with consciousness, such as self-awareness, intention and emotion, are simulated rather than realized, for example Robot K-456 (1964) by Nam June Paik and Shuya Abe (Kac, 1997), Helpless Robot (1987) by Norman White (White, 1987), and hitchBOT (Smith and Zeller, 2017). I would propose that these works and stories about sentient robots stem from contemplation of the limits of human technological agency and the hazards of transgressing what has been “designed” by nature. To embark upon the project of building a robot with artificial consciousness would convert our speculations and apprehensions into design problems and inaugurate an entirely new domain in the arts concerned with the production of autonomous creativity and the deliberate craft of human-AI culture.

One promising direction for this project is Gallese's (2017) bio-cultural approach to art and aesthetics, which is grounded in embodied cognitive processes.

The body literally stages subjectivity by means of a series of postures, feelings, expressions, and behaviors. At the same time, the body projects itself in the world and makes it its own stage where corporeality is actor and beholder; its expressive content is subjectively experienced and recognized in others (p. 181).

The material presence of a technical implementation of consciousness, allows us to confront the physical constitution of sentience. The presence of such a lively thing, as something that must be engaged spatially and socially, automates a tacit understanding of the physical constitution of our own experience of consciousness. There are, of course, other pathways to understanding the physical nature of human consciousness, or the illusion of consciousness, for example through scientific explanation but, for art, it is the presence of the aesthetic object that convenes experience and understanding.

### 1.2. GS

As a cognitive roboticist, I try to make machines come alive. Consciousness is one the most profound aspects that characterize us as human beings. Whether and how conscious machines that are aware of themselves can be created is an actively debated topic also in the robotics and artificial intelligence communities. Consciousness and self-awareness are, however, ambiguous terms and numerous theories about what constitute them have been proposed. A phenomenological account of consciousness has recently re-gained vigor in philosophy and brain sciences, which focuses on a low-level, pre-reflective aspect of consciousness: the *minimal self* (Gallagher, 2000; Metzinger, 2020). *Pre-reflective* stands for something that is experienced *before* rationally thinking about it, and mainly relates to the perception of our own body and the feeling of being in control of our own movements. This aspect of consciousness is perhaps the most easily accessible in terms of experimental exploration and quantification, and a number of measures and behavioral paradigms have been proposed in the literature (see Georgie et al., 2019 for a review). Empirical research supports the idea that such low-level subjective experiences rely on self-monitoring mechanisms and on predictive processes implemented by our brains.

Robots share similar characteristics with animals and humans: they are embodied agents that can act and perceive what is happening around them. Complex behaviors and internal representations can emerge from the interaction between their embodiments, the environments they are situated in, and the computational models implemented in their embedded computers. Building, monitoring, and analysing them, may provide insights in the understanding of different aspects of cognition, of subjective experiences (Schillaci et al., 2016; Lang et al., 2018), and of consciousness (Holland and Goodman, 2003; Chella et al., 2019).

## 2. What Are the Ethical Issues?

### 2.1. DHS

The viability of artificial consciousness is often conceived as dependent upon the development of artificial general intelligence (AGI), consciousness regarded as an emergent property of general intelligence. Although this is certainly the case in the evolution of consciousness in human beings, there is no reason to suppose that consciousness will come along for the ride in the development of AGI. The association between general intelligence and consciousness also leads some to assume that artificial consciousness has similar development challenges. This may not be the case, and we won't know without separating the project of artificial consciousness from the project of artificial general intelligence.

The model of consciousness one proposes to implement affects the formulation of an ethics of artificial consciousness. The contingent mapping of ethics to models of consciousness can be organized around the themes of suffering, moral obligation, and alignment.

A proposal distinguishing suffering from pain in human beings, regards suffering as a type of avoidance or resistance to the experience of pain, which perversely amplifies and prolongs the painful experience. Suffering in this view implicates self-knowledge and the role of language in reflecting upon and abstracting experience.

A specific process is posited as the source of the ubiquity of human suffering: the bidirectionality of human language. Pain is unavoidable for all complex living creatures, due to the exigencies of living, but human beings enormously amplify their own pain through language. Because verbal relations are arbitrarily applicable, any situation can “remind” humans of past hurts of all kinds. In nonverbal organisms, only formally similar situations will perform this function (Hayes, 2002, p. 62).

The problem of suffering in artificial consciousness as described by Metzinger (2018) derives from the assumption that consciousness and, in particular, a phenomenal self model, underwrites the capacity for suffering in human beings. Metzinger reasons that an artificial consciousness possessing human-like phenomenological models will have the potential to suffer as a result of poor or malicious design, thus it would be immoral to create an artificial consciousness. And worse, our copy-paste technologies would allow unlimited multiplication of suffering artificial patients. This is a challenging argument and one that yields some interesting questions when explored. For example, arguments for the avoidance of suffering are not reserved to the artificial. Is it not also an anti-natalist recommendation against human procreation? We should not have children by this account. Such arguments run contrary to the optimistic disposition of the majority of humankind and downplay our capacity for creative problem-solving in the face of novelty.

The moral implications of the claim that consciousness entails suffering varies depending upon whether this is a correlational or causal claim and how we think of suffering in relation to existential threats and physical damage. If what we call suffering is how the brain represents perceived threats and actual damage to our bodies, then consciousness is merely correlated with suffering. The absence of consciousness does not remove actual threats, and nor does it obviate real damage to human or animal bodies. However, the psychological nature of suffering appears to exceed this reductive correlation, particularly the types of suffering associated with remembrance, attention, and anticipation.

Psychological suffering entails attending to mental representations of pain, deprivation, revulsion, grief, anxiety, fear, and shame. Here one finds an interesting overlap between the role of mental representations and the claim that suffering might result from poorly designed artificial consciousness. Is not human suffering also a design problem entailing responses to accidents and surprises, the behavioral decisions of self and others, and our cognitive habits of representation? For example, Buddhist contemplative practices, while not claiming to resolve the causes of suffering from actual threats and real damage that come with our physical mortality, do attempt to re-frame the psychological experience of suffering through compassion, observation, and attentional training (Yates et al., 2017). Why would we not include criteria for psychological framing in the design of artificial consciousness? Metzinger does propose an applied ethics for the limited design and development of consciousness technologies, hinging upon the question, “What is a good state of consciousness?” (Metzinger, 2009).

Bryson's argument against moral obligation to machines (Bryson, 2016) also responds to the problem of multiplication of artificial patients. Bryson is pragmatic about the scope and scale of problems confronting humanity and our limited capacity to reserve care and resources to the needs of humans and animals, rather than robots, in the present and near future. Bryson does, however, consider that artificial consciousness may have creative application within the arts (Bryson et al., 2017). The latitude for experimentation with artificial consciousness within the arts may be justified by the voluntary participation of arts audiences in low-risk settings where fictions are expected.

To the extent that consciousness or, at least, the user-illusion of consciousness and self, have come to be associated with autonomy, Dennett (2019) argues that these features, in the absence of human vulnerability and mortality, would render an artificial consciousness indifferent to human values. The technical immortality of the artificial consciousness, its copy-paste methods for reproduction, and its on-off-and-on-again resistance to “death,” certainly divide the machine bearers of consciousness from the human bearers, according to susceptibility to threat and damage. But this difference does not necessitate misalignment. It is possible that the resilience of artificial consciousness in the face of existential threats has something to teach us about the design of our own experiences in the context of mortality. For example, an effectively immortal artificial consciousness may not be subject to the limits of imagination associated with our lifespan horizons, for example by engaging in counterfactual thinking conducive to the welfare of multiple generations of humanity into the future.

### 2.2. GS

Implementing conscious machines would raise, indeed, different ethical concerns. Should they be considered as objects or as living agents? Studies have shown that simple social cues already strongly affect our views of robots. For instance, people refuse to turn off a small humanoid robot when it is begging for its life (Horstmann et al., 2018), or feel the destruction of a robot—as your *hitchBOT* taught us—morally wrong (Smith and Zeller, 2017; Fraser et al., 2019).

Should conscious machines have moral competence? Making moral decisions may require empathy with pain, suffering and emotional states of others (Wallach et al., 2011). Is building conscious robots that undergo pain and suffering ethical itself? As you pointed out, the moral implications of creating suffering artificial agents, as well as of claiming that consciousness entails suffering, may vary also depending on whether we think of suffering as a mere physical damage or as a higher mental representation of experiences of negative valence, perhaps over a longer time scale.

How can we assess whether robots could go through pain and suffering, though? Even the detection and assessment of pain in animals and insects is problematic. Animal scientists have been trying to define concepts and features that can be used to evaluate the potential for pain in vertebrates and invertebrates—to name a few: the possession of nociceptors, the existence of neural pathways from nociceptors to the brain, the capability to avoid potentially painful stimuli through learning, and so the like (Sneddon et al., 2014).

Recent accounts propose that the experience of pain, as well as subjective and emotional experience, results from a perceptual inference process (Seth et al., 2012; Pezzulo, 2017; Kiverstein et al., 2019). This would explain, for instance, how pain perception seems to be affected not just by physical damages but also by past experiences, expectations and emotions (Garcia-Larrea and Bastuji, 2018). I believe that modeling these processes in robots—and integrating them within a bigger framework where behaviors are driven by different types of imperatives and goals—may help in shedding light on the nature and valence of pain, suffering, and consciousness in humans.

## 3. What Is Required for an Artificial Consciousness?

### 3.1. DHS

A naturalist theory of consciousness necessitates an evolutionary explanation of how simple organisms could evolve complex minds capable of the type of intelligent and reflexive cognitive features we associate with subjective experience. One type of evolutionary explanation proposes that consciousness arises spontaneously given some sufficient degree of complexity and integration in the information processing capacity of a biological, or indeed, a physical or technical system (for example, see Tononi, 2012). Proposing an informational approach that is tightly bound to biological life, Damasio (2012) considers the adaptive advantages of a successive stages of evolving self-modeling processes: the protoself representing vital information or primordial feelings about the body and status of the organism, the core self representing information about its interactions with other organisms, objects, and environments, and the autobiographical self comprised by complex representations combining core self and protoself with memory and future simulation. Features of consciousness associated with the autobiographical self have evolved, perhaps uniquely, in humans coincident with language and culture: “Consciousness in the fullest sense of the term emerged after such knowledge was categorized, symbolized in varied forms (including recursive language), and manipulated by imagination and reason” (Damasio, 2012, p. 182). An information-based theory of consciousness would need to process, integrate, and resolve low level incoming information with these higher-order predictive representations. Ultimately we would look to neuroscience for plausible mechanisms and implementations that integrate bottom-up and top-down information, for example Dendritic Information Theory (Aru et al., 2020).

While all naturalist theories of consciousness are equal in their status as provisional, rather than generally accepted scientific explanations, the pragmatic aim of building a synthetic consciousness recommends against the most speculative of these theories at this time, including quantum theories of consciousness (Hameroff and Penrose, 1996) and panpsychist assertions that consciousness is a fundamental (yet currently undetected) physical feature of the universe (Goff et al., 2001). I am suspicious of theories of consciousness, hijacking the anthropomorphic principle, that begin with the assertion that since we live in a universe where consciousness exists, it must therefore be a fundamental feature of the universe. Imagine replacing “consciousness” with “duck-down duvets” and you will see the troubles piling on.

This leaves in place a candidate group of information theories of consciousness that attempt to model brain-based biophysical information processes in a variety of framings, including lower level theories, which ground explanations in neural processes, and higher order theories emphasizing mental representations. A naturalistic account of consciousness maintains that phenomenal consciousness is an effect, or result, of brain functions and mental representations. These can be accounted for in higher-order cognitive theories that explain consciousness in terms of causal role, having a function in an architecture of mental processes and intentional contents. Mental states that are considered to be phenomenal consciousness “are those states *that possess fine-grained intentional contents of which the subject is aware*, being the target or potential target of some sort of higher-order representation” (Carruthers, 2016).

Thagard (2019) employs a “three-analysis” using exemplars, typical features, and explanations, to approximate a pragmatic definition of consciousness. What are typical, or broadly accepted examples of consciousness, what features do we associate with consciousness, and how is consciousness used in explaining other phenomena? Exemplars of consciousness are sensory perceptions and perceptions of pain, emotions, thoughts, and self-awareness. Typical features of consciousness include experience, attention, wakefulness, and awareness. Consciousness figures in explanations of voluntary behavior, self-reports, and wakefulness (Thagard, 2019, p. 159–160). To complete a list of ingredients for consciousness that we could use as a design specification for an artificial consciousness, I would add features identified by Metzinger (2009), such as an integrated self and world model that is continuously updating and some kind of temporal icon to provide a locus of first-person perspective in the flow of experience over time—a *now*.

The question “What causes us to report having conscious experiences?” sets aside any substantive claims about consciousness as some special kind of “stuff.” This is the research question proposed by Graziano (2016, 2019) and one which is broadly consistent with information-based illusionistic theories of consciousness (Dennett, 1991, 2016; Frankish, 2016): “To understand consciousness, we need to look for a system in the brain that computes *information about consciousness*—about its properties and consequences” (Graziano, 2019, p. 77–78). I assume consciousness to be a subset of the total of cognitive processes of the brain and body and find it plausible that the experience of consciousness consists of a reductive, and likely predictive, representation of the brain's attentional activities and intentional contents, or an attention schema (Graziano, 2018; Graziano et al., 2020). Here, it is important to highlight controversies about the nature of attention, in particular the attempted distinctions between attention, intention, and awareness, which might be more usefully subsumed under the concept of cognitive “selection” (Hommel et al., 2019).

The attention schema might also serve as a temporal icon, providing an ongoing, stable sense of presence, or “now,” in the brain's continuous updating of sequential selections. The representation of a “now” would rely upon event driven processes to mark time. The sources of events in body/brain system are attentional shifts stimulated by either mindwandering or environmental inputs, or possibly interoception of the autonomous rhythms of heartbeats and respiration. Regardless of source, an abstract representation of event driven perceptions would form the contents of type of fleeting *memory of the present* from which a sense of the immediate present, or “now” is abstracted (see also fragile short term memory in Block, 2007, 2011). In this configuration, short term memory provides a gestalt representation of the now; it feels rich, but in much the same way that a visual scene appears to be rich and complete in its detail despite its fragmentary construction by the visual system.

In fact, gestalt effects typical of visual perception, seem to be a good analogy for the phenomenology of consciousness, its feel of ineffable wholeness and ubiquity arising from piecemeal cognitive processes giving the predictive illusions of closure, similarity, and continuity. Assuming that consciousness is a reductive subset of the total of the brain's cognitive processes, a naive feature of cognitive impenetrability is required for consciousness to maintain and utilize a model of a durable observing self that believes it has global and holistic access to, and possession of, the moment-to-moment contents of experience. This naiveté is central to being a subject of conscious experience (Metzinger, 2009; Graziano, 2018; Graziano et al., 2020).

I have assembled the following table of proposed variables contributing to the phenomenology of consciousness from the ideas and literature cited above. These can be variables can serve as design criteria for an artificial consciousness. I have simplified, in some cases, by collapsing several variables under one label.

This list of variables in Table 1 could be used as a guide to the features of an artificial consciousness in a robot.

**Table 1:** List of variables contributing to reports of conscious experience.

**Variable**	**Description**
Body	A physical implementation with optimal duration or homeostasis. Since we are modeling a naturalist explanation of conscious experience, a body or physical implementation is required. Information is substrate independent, nevertheless, it requires a physical form to do something. Homeostasis is added to provide a needed value to animate the body and to distinguish salient information.
Wakefulness	Variable states of responsiveness or arousal, for example: from comatose, to dreaming, to vigilance. A minimal level of responsiveness is a pre-condition for having conscious experience.
Action	Capacity to cause changes in physical domain, including cognitive domain (information, while substrate independent requires physical implementation).
Perception	Mechanisms for sensing and representing physical domain, including cognitive domain.
Searchable memory	Mechanism and processes for short and long term retention and retrieval of representations.
Integrated self and environment model	Updatable reductive, abstract representations of “I” and “me,” “my body,” character, personality, narrative, and counterfactual self. Updatable reductive, abstract representations of physical body, others, environment, physics, and the arrow of time.
Integrated attention, intention, and temporal schema	Updatable reductive, abstract representation of perceptual attention, and intentional status. An iconic representation marking the present moment in a sequential flow of events, providing an updatable locus of perspective vis-a-vis intentional representations.
Language	Semantic and linguistic representation to communicate reports of conscious experience.

### 3.2. GS

I tend to focus on low-level phenomenological aspects of consciousness. Contemporary phenomenologists (Zahavi and Parnas, 1998, 2003; Gallagher, 2006) argue that the most basic level of self-consciousness is the *minimal self*, i.e., “the pre-reflexive point of origin for action, experience, and thought” (Gallagher, 2000). Some scholars (see Zahavi) claim that the minimal self precedes any social dimension of selfhood, while others (Higgins, 2020) see this minimal form of experiential selfhood in humans as equiprimordial with socially constituted experiences. Primitive forms of sense of self developed in early infancy have been proposed to crucially rely on caregiver-infants close embodied relationship (Ciaunica and Fotopoulou, 2017; Ciaunica and Crucianelli, 2019), which allow the developing organism to further mentalize its homeostatic regulation of interoceptive signals (Fotopoulou and Tsakiris, 2017).

Higher-order theories of consciousness explain subjective experience throughout the cognitive ability of being aware of one's own mental states (see Lyyra, 2010 for an interesting review). Whereas higher-order theories of consciousness can be useful in differentiating forms of self-awareness, they do not offer a clear account of how it bootstraps and of how “infants or animals can undergo phenomenal experience without being aware of such phenomenal states” (Lyyra, 2010). I think that a more pragmatic approach to the implementation of a *developing* artificial consciousness would better start from more minimal forms of experiential selfhood, addressing low-level phenomenological aspects of consciousness.

Developmental psychologists and brain scientists have been seeking links between cognitive development and the experience of the minimal self. Studies showed that newborns are systematic and deliberate in exploring their own body and the consequences of their own actions, suggesting the gradual formation of causal models in their brains (Rochat, 1998; Rochat and Striano, 2000). Motor knowledge and proto-representations of the body seem to be forming already during pre-natal developmental stages (Zoia et al., 2007). Paradigms for measuring body awareness and agency attribution in infants (Filippetti et al., 2014; Filippetti and Tsakiris, 2018), as well as in adults (Shergill et al., 2003; Ehrsson et al., 2004), can be also found in the literature. As mentioned above, caregiver-infants close embodied relationship seems to support the development of primitive forms of a sense of self (Ciaunica and Fotopoulou, 2017; Ciaunica and Crucianelli, 2019).

These studies indicate emergent conscious phenomenology already during early developmental stages. But what is driving this process? What are the computational and behavioral prerequisites that would let this emerge also in robots? If we take a developmental standpoint, some of the variables that you suggested in Table 1 may be appearing at later stages of development, and others may be more intertwined. For instance, language may be not essential in early developmental stages of consciousness. Developmental psychologists measure subjective experience in infants through non-verbal indicators, e.g., looking time to visual stimuli, hemodynamic response measured through brain imaging techniques, number of movement units of their limbs, etc. An integrated self-representation seems to emerge throughout embodied interactions.

Experience affects perception, as well: what our brain perceives seems to be shaped by prior beliefs and expectations, according to the predictive brain hypothesis (Clark, 2013). The Free Energy Principle (FEP) (Friston, 2009, 2010) brings this forward, suggesting that brain functioning can be explained under the single imperative of minimizing prediction error, i.e., the difference between expected (or predicted) and perceived sensations (Pezzulo, 2017). Recent research posed a link between predictive processes, curiosity and learning (Oudeyer et al., 2007), and emotional experience (Kiverstein et al., 2019). According to these proposals, biological systems not only track the constantly fluctuating instantaneous errors, but also pay attention to the dynamics of error reduction over longer time scales. Interacting with the environment as part of epistemic foraging may generate more prediction error, but nonetheless may feel good for the agent. I find these studies extremely interesting and I feel that these processes may have a role also in conscious experience. Analysing the rate at which those errors are being reduced or increasing over time may provide insights about emotional engagement in humans and its implementation in artificial system. In a recent study with Alejandra Ciria and Bruno Lara, we showed that linking prediction error dynamics, emotional valence of action and self-regulatory mechanisms can promote learning in a robot (Schillaci et al., 2020a). The generative models that realize adaptive behaviors in biological systems may be driven by different drives (Pezzulo, 2017). Self-regulatory mechanisms should be also taken into account in the development of an artificial consciousness.

### 3.3. Complementary Strategies

In summary, two complementary approaches to the challenge of building an artificial consciousness are taken here. DHS tends toward a higher-order theory of consciousness, focusing on the importance of mental representations, such as primordial to complex self models and their contribution to conscious phenomenology. GS takes a lower-level approach which seeks to explain phenomenal, minimal self-experiences by means of embodied and computational processes, such as predictive processes. He presumes that embodied interactions with the world and with other individuals support the gradual formation of internal models and representations, ultimately allowing reflective conscious phenomenology at later stages of the developmental process.

Both DHS and GS converge on naturalist, developmental and brain-based explanations of the evolution and emergence of conscious experience.

## 4. How to Begin?

### 4.1. DHS

Assuming a higher order theory of consciousness, the variables that contribute to conscious experience need to be modeled in an architecture of representations derived from fine-grained neural activity. How the brain's neural representations are encoded and related in such an architecture is an open question. As I understand it, approaches to encoding and decoding higher order representations can proceed by either attempting to imitate what the brain does when it construes complex representations, or by following computational methods that might achieve similar results by different means.

I am not sure where I first encountered the analogy (maybe Edwin Hutchins?), but I like to think of this choice of computational algorithmic vs. implementation level approaches as *fish vs. submarine*. If you want to design and build something that can swim underwater you could try to manufacture an artificial fish in all of its detail, or you could build a submarine. The analogy helps me think about the advantages and disadvantages of the two approaches for artificial consciousness. Building a fish will produce the desired result eventually but might also consist in wasted research and development effort in the reproduction of trivial, or irrelevant features, such as how to achieve the unique variation in the colored speckles of trout skin. On the other hand, building a submarine may result in overlooking critical fish features, such as the friction drag reduction of the scales on trout skin. Ideally, an artificial consciousness designer would avail of the function approximating approach of submarine (computational) design, while drawing inspiration from the salient features of fish (brain) design.

**Figure 1:**
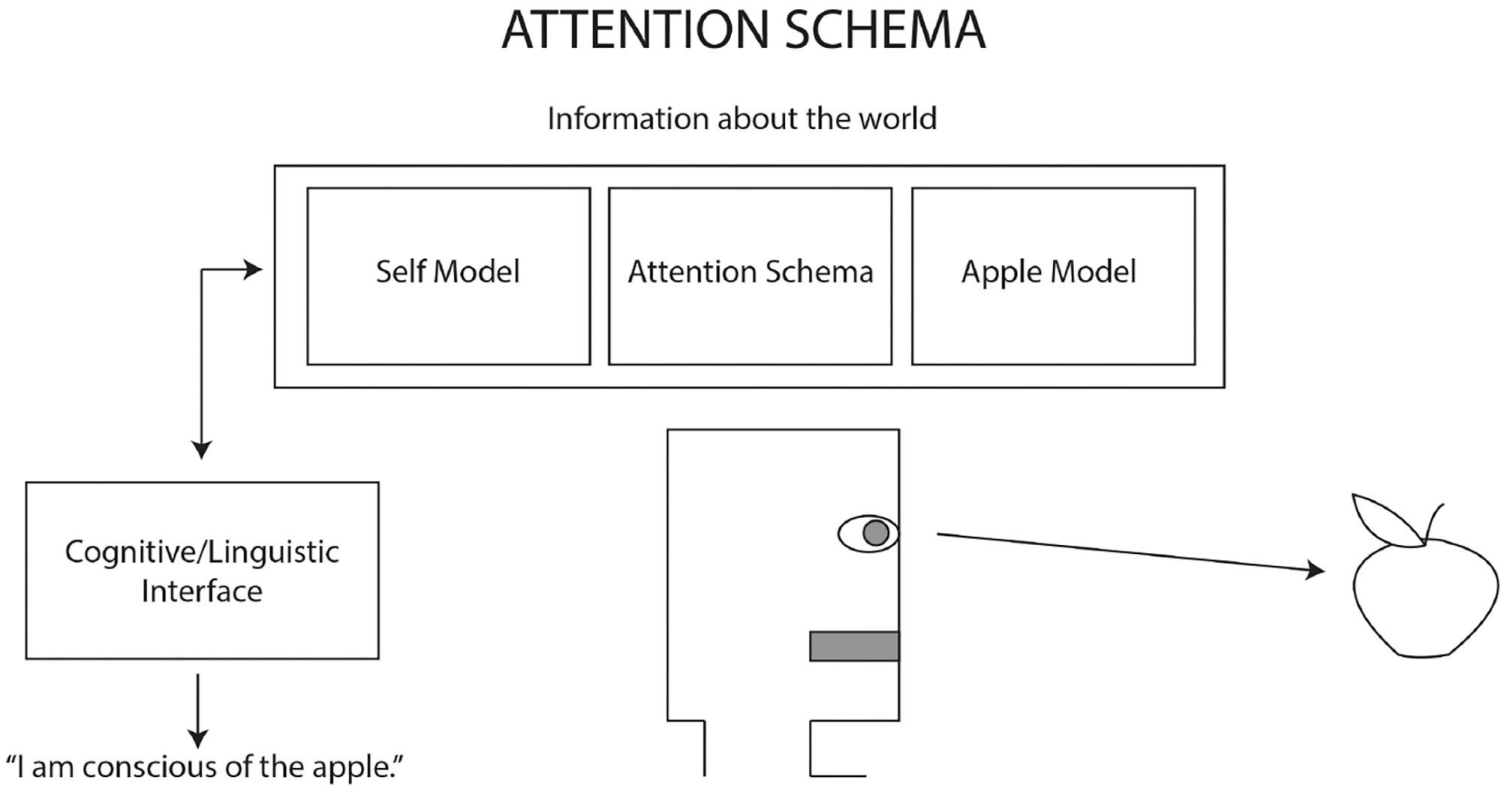
Adapted from Graziano (2019, p. 174). The attention schema incorporating cognitive features of objective awareness, cognitive access, and self-model.

For describing the functions and integration of cognitive systems giving rise to conscious experience, the attention schema in Figure 1 (Graziano, 2016, 2019; Graziano and Webb, 2018; Graziano et al., 2020) for building artificial consciousness looks like a good place to begin. Graziano and Webb (2018) propose a design sketch of the key features required to build artificial consciousness. These include a layered set of cognitive models beginning with (1) objective awareness of something, such as a perception of an apple, (2) cognitive access, or an information search and retrieval capability with a linguistic interface that can report information on the machine's internal models, (3) a self-model, or information about the machine's body, history, capabilities, and (4) an attention schema which integrates the layers of objective awareness and self-modeling information and is able to report this integrated relationship. The attention schema represents the machine's current allocation of computing and sensor resources to the contents of its objective awareness and the relation of these intentional contents to the self-model.

The attention schema layer is also where phenomenological features are implemented. For example, the sense of subjective awareness as something that feels internal and approximately spatially anchored to the self-model and the sense that the contents of awareness are something possessed by the self and available to be acted upon by the self. A machine with the proposed layered cognitive features of object awareness, cognitive access, self-model, and attention schema, should be able to report, “I have mental possession of an apple” (Graziano and Webb, 2018, 294).

Most importantly, the attention schema is also naïve about its own construction. Because the schema is only able to report on information that it has access to, and it does not have access to information about its own coding and hardware functions, the schema is *transparent* to itself; it suffers from cognitive impenetrability. Such a conscious machine could have a parallel set of information processes that are able to objectively monitor and report on how the whole system is put together “how the representational models are constructed—under the hood” (see Holland and Goodman, 2003 for a discussion of this transparency). This would be a machine that has one system for naïve subjective awareness and another system for objective analysis, very much like the much maligned homunculus philosopher of mind ☺.

An artificial consciousness would also require some overarching objective to guide its values for information seeking and constructing salient representations. For example, the varieties of information that a human self-model abstracts, such as physical body, sense of agency, and social status, are finely tuned to prioritize genetic replication. Defining and declaring these orienting values for self modeling in an artificial consciousness involves design decisions with moral and ethical implications (Metzinger, 2009, 2018; Dennett, 2019), thus “survival and/or replication” might not be the wisest choice for arbitrarily assigned values to guide the behavior of our artificial consciousness. A more genteel and human-compatible objective for a robot with artificial consciousness might be “to learn and model knowledge about human consciousness” with some safeguards to ensure that the robot's information seeking behaviors are the result of voluntary human-robot interactions and decidedly passive and observational in execution. Such an objective would necessitate modeling the values that shape human consciousness, providing an overlapping domain of aligned objectives between sentient machines and human beings. Adding values by design suggests that we are engaged in building a hybrid symbolic and deep learning model, one that relies upon both assigned and learned values.

**Figure 2:**
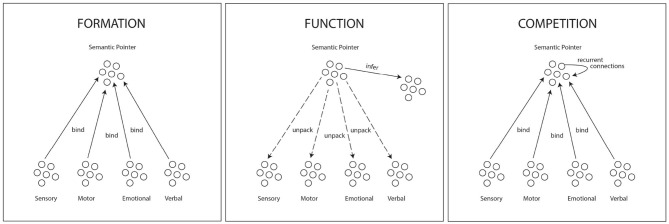
Adapted from Thagard and Stewart (2014, p. 74–76). “Semantic pointers function to provide inferences by virtue of relations to other semantic pointers and can also unpack (decompress) into the sensory, motor, emotional, and/or verbal representations whose bindings formed the semantic pointer.”

Given the gap that exists between the type of fine-grained unstructured data generated by the robot's sensors and the complex representations required for an attention schema, we need a computational method for building complex representations. Semantic pointer architecture or SPA in Figure 2 (Eliasmith, 2013; Thagard and Stewart, 2014; Thagard, 2019), models encoding of data into the type of layered cognitive models required in the attention schema. SPA models how multiple sources of granular information acquired in networks of lower level sensory and motor neurons can be formed into more complex representations, binding neural networks through pointers. SPA models how representations function by decomposition, or unpacking, to their constituent information networks and how neural network representations can point to or infer other complex representations. Competition among semantic pointers through recurrent connections among neurons provides a process which could support gestalt cognition, shifting attention, representing changes in experience, and mindwandering.

Assuming that we have, in the attention schema, a plausible theory of artificial consciousness, and a practical method for encoding and decoding neural networks to achieve its constituent cognitive models, what remains is to create an experimental design for the robot based on causal modeling and evidence testing.

**Figure 3:**
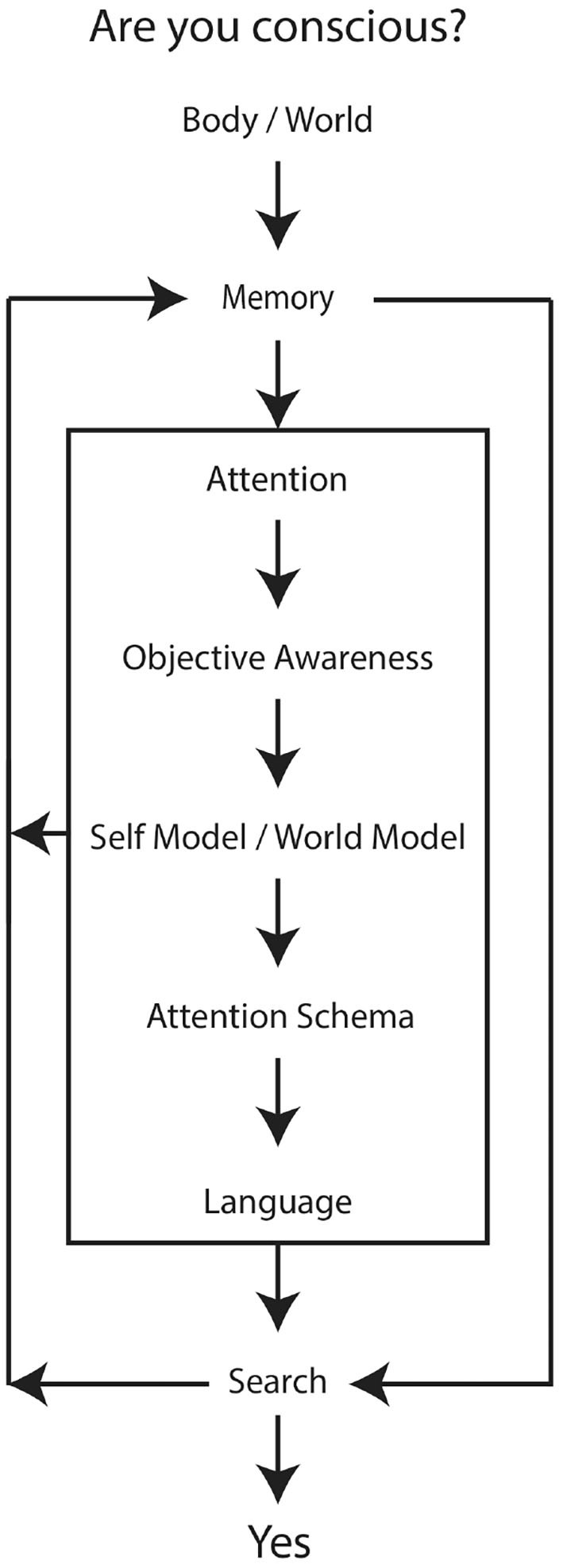
Causal Diagram for Artificial Consciousness in a Robot. The arrows indicate direction of cause and effect. Reverse direction indicates “listens to,” for example the self-model/world model listens to the objective awareness function, which in turn listens to the attention function. Attention, objective awareness, self and world models, the attention schema, and language also listen to memory and, in turn, shape memory. Downstream of body/world, all functions are proposed to be constituted by a searchable (unpackable) semantic pointer architecture.

A causal diagram (Pearl and Mackenzie, 2018) would indicate what causes the robot to have, and report, conscious experience. The diagram should incorporate the variables, or combinations of variables, listed in Table 1, all of which are explicit or assumed in the attention schema, as well as some type of intervention to activate the chain of cause and effect (see Figure 3). In this case the intervention is the question, “Are you conscious?,” posed to the robot. This is, in all likelihood, spectacularly wrong-headed, but I am more than happy to start with “wrong” so that I can enrich my life by starting interesting arguments with friends.

Body, world, and memory are variable sources of intentional relations. The robot's attention may be directed toward information coming from its body, its environment, and its memory, which would include successive updating loop of self models (proto, core, and autobiographical). Attention information supplies objective awareness the substance of consciously accessible perceptions and interoceptions. All informational contents are bound together in a semantic pointer architecture, such that domain specific information, like the body model, is actually composed of inferences and predictions from its constituent neural networks in the architecture. The neural architecture supporting the attention schema contributes unpackable lower-level information from cognitive processes related to the body, the self, the world, objective awareness, memory, and attention. There is no binding problem in this model of consciousness because the attention schema is a gestalt-like prediction generated by this architecture. Memory and the informational contents of objective awareness inform the self and world models. The profile of objective awareness, which is constituted by a variable emphasis of the combined subjective and objective models, informs the attention schema. The schema, from a phenomenological perspective, is searchable because it is taken into short term memory and it may be queried and decomposed to its constituent world, or object, and self models. A short term memory loop may entail a type of buffering memory, with a fade-in prediction and fade-out memory gradient centered on an abstract representation of “now”—this would provide an always-advancing-into-the-future temporal icon upon which can be hung the “what it feels like” of conscious (hetero)phenomenology.

### 4.2. GS

Graziano's higher order theory of consciousness has some aspects that sound plausible to me, others rather more problematic. For instance, the proposal that conscious experience requires a model of the self, which would comprehend low-level bodily aspects and high-level autobiographical aspects of the self (Graziano and Webb, 2018), reminds me of Gallagher's distinction between minimal self and narrative self (Gallagher, 2000). As argued before, phenomenology of the self seems to emerge already during early infancy, likely before more complex, say autobiographical, models of the self develop.

**Figure 4:**
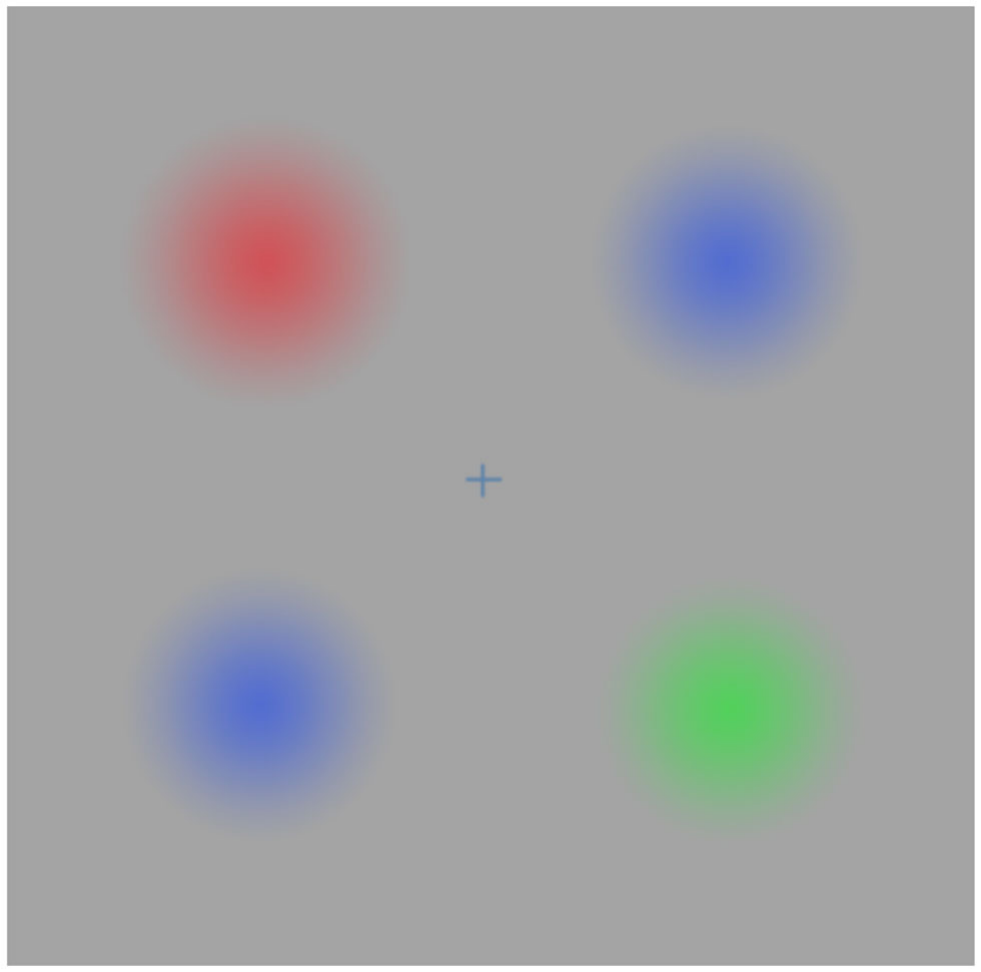
Adapted from Parr et al. (2019). Troxler fading: when fixating the cross in the center of the image, the colors in the periphery gradually fade until they match the gray color in the background; when saccadic exploration is performed, colored blurred circles become visible.

Graziano also suggests that our brains maintain internal models of objects, and argues about the need of an objective awareness component: when sensory information about an object is available and is processed, the machine becomes objectively aware of that object. I subscribe to the idea that our brain makes up internal models of the world, but perception seems to have a more inferential, hypothesis testing nature than previously thought (Clark, 2013). This would already assign a subjective flavor to our awareness of the external world. Perception can be influenced by many other things, even by the presence or absence of action (see, for example, Troxler fading illusion reported in Parr et al., 2019 and in Figure 4).

Another comment is on the cognitive access component and the linguistic interface that—although not essential (Graziano and Webb, 2018)—would make the experimenter able to query the machine. However, different levels of consciousness can be attributed to animals and people from few behavioral features, without the need to engage in a conversation. I would thus explore—before the linguistic interface—which robot behaviors could induce us in the attribution of consciousness.

I would also look at more robust methods to quantify subjective experience. In a recent paper, we discussed different paradigms and measures used in cognitive and brain sciences, and reviewed related robotics studies (Georgie et al., 2019). What would constitute a successful demonstration of artificial consciousness (Spatola and Urbanska, 2018)?

This also relates to the central element of Graziano's theory: the attention schema. Graziano suggests that the machine can claim it has subjective experience “because it is captive to the incomplete information in the internal models”—i.e., the models of the self and of the object, through an internal model of attention (Graziano and Webb, 2018). Subjective awareness of something would be thus “a caricature of attention.” As he claims, if a machine can direct mechanistic attention to a specific signal, and if the machine has an internal model of that attentional state, then the machine can say that it is aware of that signal. I recognize that attentional processes may have an important role in conscious experience, as well as in perception and action, but this conclusion sounds too simplistic to me. Moreover, how would such an attention schema be concretely implemented? I find interesting an account that comes with the active inference proposal (Feldman and Friston, 2010), where attention is viewed as a selective sampling of sensory data that have high-precision in relation to the model's predictions. In a way, this is deeply intertwined with the agent's internal models, more than—as it sounds to me—as in Graziano's model. These comments would apply also to your causal diagram.

I find the semantic pointer architecture (SPA) interesting. Similar works on grounding complex representations on multi-modal experience can be found in the developmental robotics literature. An example is the Epigenetic Robotics Architecture (ERA) (Morse et al., 2010), used for the acquisition of language and body representations in a humanoid robot. ERA self-organizes and integrates different modalities through experience. I have also studied similar models for the incremental learning of internal models (Escobar-Juárez et al., 2016; Schillaci et al., 2016), where representations were grounded on integrated motor and sensor maps, similarly to SPA. I also investigated how predictive capabilities could emerge from such representations, and how prediction errors could be exploited as cues for self-perception (Schillaci et al., 2016). Similar processes are thought to be involved in minimal self experiences.

### 4.3. DHS

As you point out, objective awareness entails prediction, but I think predictive processing is consistent with the attention schema model through input of salience values and prior conditioning and the role these play in perceptions associated with objective awareness. Additionally, objective awareness in AST is not exclusively reliant upon environmental inputs and gross physical actions, interoception and memory also supply inputs to objective awareness.

On the issue of verification of consciousness, admittedly the approach taken by AST of simply asking the robot if it is conscious seems facile (as I scurry off to read your papers). But I believe this superficial approach has merits that are specifically relevant to artificial consciousness and the AST model. In the AST model, human consciousness is an informationally impoverished representation of attention; representations of objects, the world, the self, and attention do not include information about the processes leading to representation in the brain. The self that claims to have conscious experience is ignorant of the neurological mechanisms that cause the claimed experience and experiencer. In this respect, it is an important evaluative tool to test for this ignorance. However, as evaluators of an artificial consciousness, we also have access to the systems of the AI that are impenetrable to itself. We can know and monitor the performance of the nested set of representations in our causal model, to see how they are engaged when the robot considers the query “Are you conscious?” In theory, we would have evaluative tools combining synthetic self-reports and quantitative measures of the systems producing the self-reports.

## 5. What Is the Role of Creativity in Artificial Consciousness?

### 5.1. DHS

The project of building an artificial consciousness engages with creativity in several contexts. First, there is the question of how synthetic consciousness will be included by artists in the materials and methods of art making. Much of contemporary art is motivated by politics, criticism, and reflexivity. While an art of artificial consciousness might become just another medium that artists may use to express these secular contents, its sentient aspirations might otherwise reinvigorate an aesthetics of existential wonder. Rather than promoting anthropocentric hubris, as some might claim, artificial consciousness confronts us with the humbling genesis of mind from matter, and the emergence of subjective experience in a non-differentiated physical field. In the case of a synthetic consciousness, our attention and critical appraisals must be directed to the form or medium of the artwork, rather than its ostensible contents. Often, in the discussion of consciousness, one encounters a division between the contents of consciousness and consciousness itself. Most artists will recognize a striking similarity between this distinction and the historic tensions between formalism (materials, methods, and ground) and representation (symbolism, reference, meaning) in art (Zangwill, 1999). The artistic engagement with artificial consciousness would constitute an unsurpassable formalism. After all, isn't consciousness the ground of all appearances and, ironically, itself an appearance?

Secondly, there is the creativity of the synthetic consciousness itself. An artificial consciousness will be an historic event in the human development and use of symbolic media, in this case, the technical investment of another kind of introspecting perspectival witness to the unfolding universe. Due to the transparent nature of its consciousness, this would be an artwork possessed of its own boredoms and uncertainties, and consequently prepared and motivated for the work of curiosity and creativity. Of course, creative functions leveraging uncertainty, such as mind-wandering behavior would require design and implementation. Mind-wandering requires the ability to combine representations in increasingly complex and novel formations and, importantly, to decompose representations to their constituent lower level representations. In this way, an artificial consciousness could travel the space of ideas, associating, assembling, disassembling, and reassembling unique proposals, in search of novel representations to satisfy its aesthetic values.

The cognitive scientist Margaret Boden describes three types of creativity: exploratory, combinatorial, and transformative (Boden, 2009). The first two types of creativity, exploratory and combinatorial, describe novel, or surprising outcomes as artists engage with either in-depth or hybrid investigations of familiar, rule-bound domains. Transformational creativity, on the other hand, constitutes “changing the rules,” or a perturbation of these domains (Du Sautoy, 2020). Such perturbation according to Du Sautoy (2020), would likely stem from a disruption of our current assumptions about the role of free will in artistic creation,

Our creativity is intimately bound up with our free will, something that it seems impossible to automate. To programme free will would be to contradict what free will means. Although, then again, we might end up asking whether our free will is an illusion which just masks the complexity of our underlying algorithmic process (Du Sautoy, 2020, p. 301).

The confrontation with artificial consciousness, with its phenomenological connotations of experience, creativity, and self-expression, might, as Du Sautoy suggests, motivate better explanations of the cognitive processes that appear to us as human creativity.

One of the projects of an artificial consciousness might be the discovery of unique aesthetic values, perhaps a sense of beauty that is salient only to the conscious machine. For example, in what ways would an artificial consciousness surprise us? Surprises of observational profundity, sensory pleasure, and narrative fulfillment, are what we have come to value in the arts, but I wonder what are the aesthetic possibilities of scientific creativity? Given the role of creativity in proposing scientific explanations and the knowledge that all scientific explanations are destined to be approximations of reality, is it possible that our artificial consciousness could use its transformative creativity to generate multiple novel, yet viable, approximations of reality, distinguished only by their aesthetics, their framing of the sublime? Science and art will converge in creative artificial consciousness.

### 5.2. GS

I agree with you that this project engages with creativity on many aspects: in the creative process of designing and building the artificial consciousness; in the new perspectives and possibilities that an artificial consciousness could open to artists; in developing conscious agents that are creative themselves.

We are not so far—I think—from having creative machines. There are examples out there of generative systems that can be used in explorative and creative processes—Google's deep dream, to name one, which is capable of generating novel visual artifacts from an initial knowledge of drawings and paintings. I believe that such systems would fit, however, within the category of “novel tools for creative people.” They do broaden exploration possibilities, but the creativity of such algorithms is very much biased by their designer, who outlines the underlying AI machinery, decides how to train them and how they should explore, and eventually selects the best generated samples. Somehow, such AIs are given aesthetic values already from their creators.

I find very interesting your idea of studying whether and how aesthetic values could, instead, develop in a conscious learning machine. I can imagine that basic aesthetic values and drives could be given a priori by the designer. Then, I wonder whether this unique sense of beauty that you mention, which is salient only to the machine, could develop throughout its lifetime. Experiences may form attitudes and interests, shape the temperament and emotional engagement in the various activities, and consequently affect the aesthetic values and creativity of such an artificial agent.

The cognitive architecture you depicted can be in part implemented with tools that are currently under investigation in robotics and AI (see algorithms generating artificial curiosity and novelty-seeking behaviors; Schmidhuber, 2006; Oudeyer et al., 2007; Schillaci et al., 2020b). I think that the gap between curiosity and creativity, here, is small. Intrinsic motivation algorithms are driven by epistemic value “which correlates to the reduction of uncertainty” of an action, but could be designed also to be driven by aesthetic value. Would this be enough to produce a machine that develops a sense of beauty?

## All Together Now…

Although many of the issues featured in our dialogue are represented in the current literature, we hope that our discussion of the creative application of artificial consciousness helps to concretize these issues.

Consciousness appears to be a subset of the whole of human and animal cognitive activity, composed of composite and layered processes, rather than a singular process or yet-to-be-discovered substance. To design and build an artificial consciousness requires beginning with and resolving low-level processes which, further on, may develop complex higher order cognitive features, such as the autobiographical self. According to the reviewed proposals, the phenomenology of consciousness in human beings features a stream of selected representations that appear to be governed by competition in the context of limited cognitive resources and adaptive pressures for decisive action. This raises the possibility that consciousness is the result of constraints that are not necessarily the case in an artificial system with extensible computing capacity in low risk settings. Must we design artificial dangers and constraints in our artificial system to promote the phenomenology of a stream of consciousness, or rather, allow for multiple parallel streams of consciousness in a single entity?

We take seriously the ethical concern for the potential of artificial consciousness to suffer but we differ on the best course of action to take in response to this concern. It is within the realm of possibility that an artificial consciousness may happen by accident, for example in the case of a self-programming AI, and therefore we conclude that the deliberate project of designing an artificial consciousness capable of ameliorating its own suffering is an important undertaking and one which is at least the shared concern of the arts disciplines.

We have discussed low-level computational and behavioral features that we believe would be needed for building an artificial consciousness but admit to the difficulty of deriving the required higher order representations. We consider embodied interactions as of fundamental importance for the incremental learning of the dynamics of perceptual causality. It is upon embodied intentional experience and attentional capacity that, since early in life, we construct beliefs and expectations about ourselves, our bodies and our surroundings, and that we define values on internal and external goals. An artificial consciousness should employ computational mechanisms that allow such constructions. We consider creativity in all its nuances as one of the main drives for such a development.

An artificial consciousness should be capable of perceiving what is novel or not, what is original or not, forming a sense of beauty throughout its ontogenetic experience. Aesthetic experience goes hand in hand with emotional experience, surprise, and expectation. We believe that generative models—with all the features that can be built around them, such as predictive processes, prediction error dynamics monitoring, and so the like—can lead to creative abilities in artificial systems and, ultimately, support them in assigning emotional and aesthetic values to activities and perceptions. An artificial consciousness or a creative predictive machine?

The prehistoric origins of art, according to the archaeologist Steven Mithen (Mithen and Morton, 1996, p. 229), stem from a fluidity of the cognitive domains pertaining to technology, nature, and social life, that allowed our ancestors to leverage symbolic artifacts for cultural development. After many centuries of speculation about sentient machines, we find ourselves in an age in which nature and social life might be fully reflected in our technology, an age in which our technology becomes a social presence. The advantages of this next-step in symbolic culture may lie in the role of consciousness plays in speculation and storytelling, and how these in turn support social cooperation and collaboration (Baumeister and Masicampo, 2010). Consciousness and the assumption of consciousness in each other through theory of mind, is the key to bridging the black boxes of internal cognitive processes we would otherwise be to each other. Human and machine socialization might benefit from similar assumptions.

**DHS**. Guido?
**GS**. Yes?
**DHS**. Are you a zombie?
**GS**. # @!!

## Author Contributions

All authors listed have made a substantial, direct and intellectual contribution to the work, and approved it for publication.

## Conflict of Interest

The authors declare that the research was conducted in the absence of any commercial or financial relationships that could be construed as a potential conflict of interest.
